# Evaluation of Inflammatory Markers in a Large Sample of Obstructive Sleep Apnea Patients without Comorbidities

**DOI:** 10.1155/2017/4573756

**Published:** 2017-07-31

**Authors:** Izolde Bouloukaki, Charalampos Mermigkis, Nikolaos Tzanakis, Eleftherios Kallergis, Violeta Moniaki, Eleni Mauroudi, Sophia E. Schiza

**Affiliations:** Sleep Disorders Center, Department of Thoracic Medicine, University of Crete, Heraklion, Greece

## Abstract

Systemic inflammation is important in obstructive sleep apnea (OSA) pathophysiology and its comorbidity. We aimed to assess the levels of inflammatory biomarkers in a large sample of OSA patients and to investigate any correlation between these biomarkers with clinical and polysomnographic (PSG) parameters. This was a cross-sectional study in which 2983 patients who had undergone a polysomnography for OSA diagnosis were recruited. Patients with known comorbidities were excluded. Included patients (*n* = 1053) were grouped according to apnea-hypopnea index (AHI) as mild, moderate, and severe. Patients with AHI < 5 served as controls. Demographics, PSG data, and levels of high-sensitivity C-reactive protein (hs-CRP), fibrinogen, erythrocyte sedimentation rate (ESR), and uric acid (UA) were measured and compared between groups. A significant difference was found between groups in hs-CRP, fibrinogen, and UA. All biomarkers were independently associated with OSA severity and gender (*p* < 0.05). Females had increased levels of hs-CRP, fibrinogen, and ESR (*p* < 0.001) compared to men. In contrast, UA levels were higher in men (*p* < 0.001). Our results suggest that inflammatory markers significantly increase in patients with OSA without known comorbidities and correlate with OSA severity. These findings may have important implications regarding OSA diagnosis, monitoring, treatment, and prognosis. This trial is registered with ClinicalTrials.gov number NCT03070769.

## 1. Introduction

Obstructive sleep apnea (OSA) is a highly prevalent sleep disorder and may soon be among the most common chronic diseases in industrialized countries [1]. Recent data from the United States and Europe suggest that between 14% and 49% of middle-aged men has clinically significant OSA [2]. Furthermore, it represents an evolving public health challenge, due to the associated impairment in quality of life (QOL) and functional capacity, alongside with the increased risk of medical comorbidities and mortality [3]. In particular, the relationship of OSA with cardiovascular comorbidity is among the areas of interest. The periodic upper airway collapse occurring during sleep in OSA patients induces chronic intermittent hypoxia, which is thought to promote cardiovascular disease through oxidative stress, sympathetic activation, and systemic and vascular inflammation [4–6].

Recent data have focused on the association between inflammatory biomarkers and OSA severity, as well as the prediction of cardiovascular events in patients with OSA [3]. Fleming et al. suggested that various clusters of biomarkers have an even greater association with OSA, representing physiologic signatures of the disorder that may have value in initial screening for OSA as well as for follow-up of therapy response [7]. There are variable biomarkers that are indices of systemic inflammation such as C-reactive protein (CRP), fibrinogen, erythrocyte sedimentation rate (ESR), and uric acid (UA) [8–14].

CRP, a major circulating marker of inflammation, mainly produced in the liver, is one of the best predictors for future cardiovascular morbidity [6, 15, 16]. CRP has been extensively studied in OSA and is often found elevated in OSA patients, especially in association with obesity, dyslipidemia, diabetes, and cardiovascular diseases [6, 17–19]. Fibrinogen, a major coagulation protein associated with inflammation, seems to be an important biomarker for cardiovascular risk [20], and its levels have been found to be directly related to apnea-hypopnea index (AHI) and arousal index (AI) and inversely related to mean and lowest oxygen saturation during sleep [21]. ESR which measures the tendency of red blood cells to aggregate is a widely used laboratory test of inflammation and directly associated with atherosclerosis [22, 23]. However, there have been only few reports on the correlation between the severity of OSA and plasma ESR levels. The results of a small retrospective study suggested that there may be a positive association between the plasma ESR level and nocturnal oxygen desaturation in obese patients [24]. Moreover, levels of ESR, high-sensitivity CRP (hs-CRP), and fibrinogen have been all found significantly higher in OSA patients compared to controls [25]. Additionally, other studies [12, 26, 27] indicated an association between serum UA levels, which is another inflammatory marker, with the presence of OSA. However, all the abovementioned markers have been studied mainly in a small number of OSA patients, in the presence of comorbidities, potentially affecting the above results.

The aim of this study was to investigate possible relationship between OSA and biomarkers of inflammation in a large number of OSA patients without comorbidities. Specifically, we aimed to assess the circulating levels of four inflammatory markers, hs-CRP, fibrinogen, ESR, and UA, in a large population of consecutively enrolled, untreated and otherwise healthy OSA patients. A secondary objective was to investigate potential correlation between these markers and clinical and polysomnographic (PSG) parameters of OSA patients.

## 2. Materials and Methods

### 2.1. Study Population

We conducted a single-center, cross-sectional study of patients who had undergone a PSG study for OSA diagnosis. Between June 2012 and June 2016, 2983 consecutive patients, who were admitted to the Sleep Disorders Center, Department of Thoracic Medicine, Medical School, University of Crete, for evaluation of suspected sleep-disordered breathing, were considered as potential recruits for this study. The exclusion criteria were as follows: refusal to participate, previous OSA diagnosis and treatment, subjects younger than 18 years, central sleep apnea syndrome (CSAS) diagnosed with PSG, known comorbidities (malignancy; diagnosis of chronic obstructive pulmonary disease; interstitial lung disease; asthma; diabetes mellitus; hyperlipidemia; hypertension; cardiovascular disease, such as coronary artery disease and heart failure; cerebrovascular disease; renal or hepatic dysfunction; hematological or inflammatory diseases; endocrine disease; and liver and gastrointestinal disorders), pregnancy, and history of narcolepsy or restless leg syndrome. We also excluded patients having active infections and those currently using steroids, testosterone supplements, DHEA, or pain medications.

**Figure 1:**
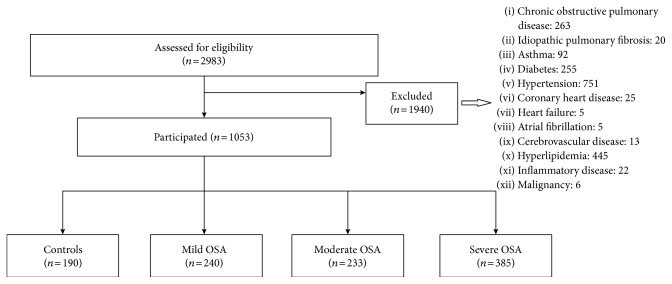
Study flow chart.

On the basis of the above criteria, 1940 patients were excluded (Figure 1). Included patients (*n* = 1053) were grouped according to AHI as mild, moderate, and severe. Patients with AHI < 5 served as the control group. All subjects provided written informed consent, and ethical approval was provided by the University Hospital Ethics Committee (approval number: 7370).

### 2.2. Initial Visit/Evaluation Data Collection

All patients underwent a detailed clinical evaluation that included age, body mass index (BMI), medical history focused on sleep-related symptoms, associated conditions and comorbidities, smoking history, and alcohol intake. Subjective daytime sleepiness was assessed by the Epworth sleepiness scale (ESS) [28]. Spirometry and arterial blood gas analyses were within the normal range in all participants, and blood pressure measurements were below 130/80 mmHg.

### 2.3. Polysomnography (PSG)

All patients underwent a single-night full diagnostic PSG study (Alice 5, Diagnostics System, Respironics, USA) according to standard techniques, with monitoring of the electroencephalogram (EEG), electro-oculogram (EOG), electromyogram (EMG), flow (by oronasal thermistor and nasal air pressure transducer), thoracic and abdominal respiratory effort (by respiratory induction plethysmography), oximetry, and body position. Snoring was recorded by a microphone placed on the anterior neck. Based on manufacturer's recommendations, equipment maintenance and calibration prior to each patient's polysomnography was performed. Polysomnographic recordings were manually interpreted over 30-second periods, by skilled staff, in accordance with the current American Academy of Sleep Medicine (AASM) guidelines [29]. The scorer was always the same person, who was blinded to the origin of the data. The determination of sleep stages and arousals was performed according to the AASM 2012 criteria, using EEG montages including frontal, central, and occipital leads. The definition of apnea and hypopnea followed the AASM standard criteria [29]. The apnea-hypopnea index (AHI), calculated as the number of apneas and hypopneas per hour of sleep, was used to diagnose OSA and assess its severity. OSA was considered mild if ≥5 AHI ≤ 15 per hour, moderate if >15 AHI < 30 per hour, and severe if AHI ≥ 30 per hour.

### 2.4. Blood Collection and Analysis

Venous blood was collected in all subjects for biomarker measurements between 8:00 and 9:00 AM, following an overnight fast, shortly after the conclusion of the overnight PSG. All venous samples were centrifuged, and serum was separated into multiple aliquots and stored at −80°C until assay. CRP levels were measured by means of particle-enhanced immunonephelometry using BN Systems (Dade Behring Inc., Newark, USA). Fibrinogen levels in plasma were measured by coagulation method. The ESR was determined by the classic Westergren method. Additionally, in the sample of blood serum, UA was measured (enzymatic colorimetric method).

### 2.5. Statistical Analysis

Results are presented as mean ± standard deviation (SD) for continuous variables if normally distributed and as median (interquartile range) if not. Qualitative variables are presented as absolute number (percentage). For comparisons between groups, a two-tailed *t*-test for independent samples (for normally distributed data) or a Mann–Whitney *U* test (for nonnormally distributed data) was utilized for continuous variables and the chi-square test for categorical variables. Correlation coefficients were calculated using the Pearson or Spearman (for nonnormally distributed data) correlation test. As independent variables, we included clinically relevant variables: age, gender, BMI, ESS, smoking history, AHI, oxygen desaturation index (ODI), AI, mean and minimum SaO_2_, and sleep time spent with SaO2 less than 90% (TST90). These variables associated with the four biomarkers at a statistical level of 0.10 were included in multivariable analysis. Multivariate linear regression analysis was used to examine any association of OSA severity (defined by AHI) with the four biomarkers, after controlling for the potential confounders that were found to be significant. Results were considered significant when *p* values were <0.05. Data were analyzed using PAWP 17.0 software (SPSS Inc., Chicago, IL).

## 3. Results

### 3.1. Patient Characteristics and Polysomnographic Findings

One thousand and fifty-three subjects (792 males, 261 females) participated in the study. Using AHI scores for classification of severity resulted in 190 controls (18%), 240 subjects with mild OSA (22.8%), 233 with moderate (22.1%), and 385 with severe OSA (36.6%). The clinical variables collected for the four severity groups are summarized in Table 1. Age, gender, BMI, and ESS score were significantly different between the four groups (*p* < 0.001). Smoking was not different between all groups although it was more prevalent in the severe OSA group compared to control and mild OSA groups (*p* = 0.036 and *p* = 0.028, resp.). As expected, AHI, ODI, mean SaO_2_, min SaO_2_, and TST 90 were worsened as the severity of OSA increased.

### 3.2. Inflammatory Biomarkers

**Figure 2:**
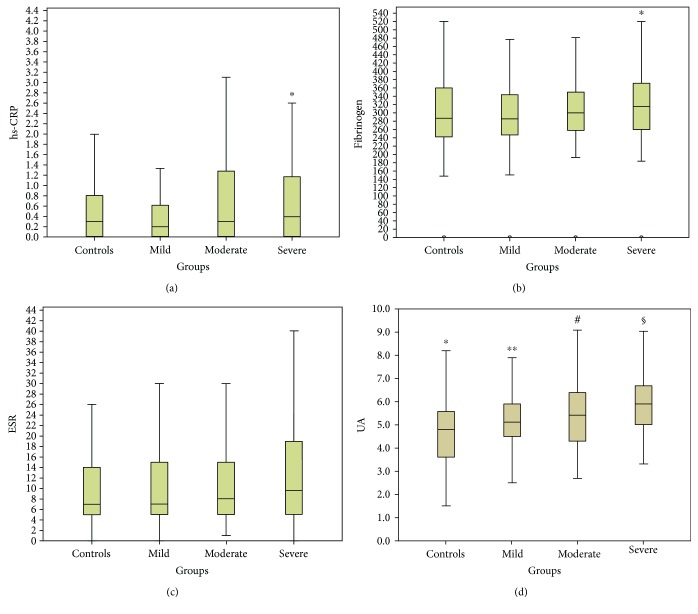
Serum high-sensitivity CRP (hs-CRP), fibrinogen, erythrocyte sedimentation rate (ESR), and uric acid (UA) are elevated in OSA patients. (a) Serum hs-CRP is found elevated only in severe compared to mild OSA patients but not in control, mild, or moderate OSA patients. ^∗^*p* < 0.05 versus mild group. (b) Serum fibrinogen is found elevated only in severe compared to mild OSA patients but not in control, mild, or moderate OSA patients. ^∗^*p* < 0.05 versus mild group. (c) There were no significant differences between all groups for ESR (*p* > 0.05). (d) UA levels are significantly different between all groups, apart from mild versus moderate groups. ^∗^*p* < 0.05 versus mild, moderate, and severe groups. ^∗∗^*p* < 0.05 versus control and severe groups. ^#^*p* < 0.05 versus control and severe groups. ^§^*p* < 0.01 versus control, mild, and moderate groups.

There was a statistically significant difference between groups in median hs-CRP and fibrinogen levels (*p* = 0.002 and *p* = 0.009, resp.). This seemed to be principally driven by the severity of OSA, since hs-CRP and fibrinogen were found elevated only in severe OSA (AHI > 30) compared to mild OSA (0.4 versus 0.2, *p* < 0.001 and 314.5 versus 296, *p* = 0.003, resp.) (Figure 2). Furthermore, UA levels were significantly different between all groups (*p* < 0.001), apart from mild versus moderate group (*p* = 0.12). There were no significant differences between all groups for ESR (*p* > 0.05).

To further analyze the relationship between biomarkers levels and OSA, we categorized these variables into high or low levels (Table 2). Hs-CRP was elevated (≥1 mg/dL) in moderate and severe compared to mild OSA patients (*p* = 0.02 and *p* = 0.008, resp.), fibrinogen was elevated (>400 mg/dL) in severe compared to moderate (*p* = 0.016), and UA was elevated (>7 mg/dL) in severe compared to control and mild (*p* = 0.001 and *p* = 0.008) as well as in moderate compared to controls (*p* = 0.036). There were no significant differences in the percentages of subjects with elevated ESR (>30 mm/hr) between all groups (*p* > 0.05).

### 3.3. Correlations and Multivariate Regression Analysis

Across all subjects, significant correlations were observed, with all biomarkers showing the strongest association with gender, BMI, and measures of OSA severity (AHI, ODI, and minimum and mean sleeping SaO_2_) (*p* < 0.001). Furthermore, all biomarkers, apart from fibrinogen (*p* = 0.12), were correlated with TST90 (*p* < 0.001). Fibrinogen was correlated with active smoking (*p* = 0.001), ESR with age (*p* < 0.001), and UA with AI (*p* < 0.001). No correlation was noted with active smoking and hs-CRP (*r* = 0.02, *p* = 0.6), ESR (*r* = −0.05, *p* = 0.17), and UA (*r* = −0.05, *p* = 0.2) levels.

Using stepwise multiple linear regression models, the effect of OSA severity on levels of inflammatory markers was measured after adjusting for the potential confounders that were found to be significant (such as gender, BMI, and active smoking). Significant associations between hs-CRP (*p* = 0.035), ESR (*p* = 0.005), UA (*p* = 0.008), and AHI were sustained even after adjusting for confounders. Although AHI did not remain a statistically significant predictor for fibrinogen, a significant association was still found between fibrinogen and ODI (*p* = 0.01). Moreover, ESR was still associated with ODI (*p* = 0.001) and min SaO_2_ (*p* = 0.01) and UA with ODI (*p* = 0.001) and mean (*p* = 0.042) and min SaO2 (*p* = 0.005) after adjusting for confounders.

### 3.4. Subgroup Analysis by Gender

**Figure 3:**
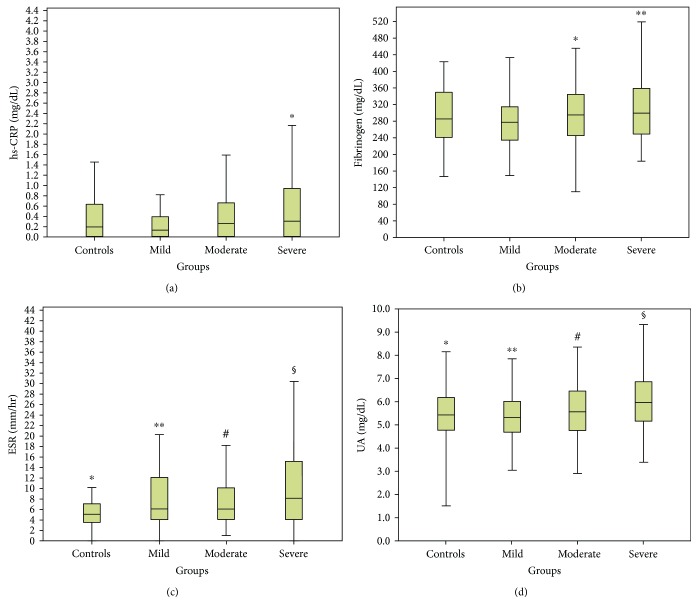
Serum high-sensitivity CRP (hs-CRP), fibrinogen, erythrocyte sedimentation rate (ESR), and uric acid (UA) in males according to OSA (obstructive sleep apnea) severity. (a) Serum hs-CRP is found elevated only in severe compared to mild OSA patients but not in control, mild, or moderate OSA patients. ^∗^*p* = 0.001 versus mild group. (b) Serum fibrinogen is found elevated in severe and moderate compared to mild OSA patients but not in control and mild OSA patients. ^∗^*p* = 0.02, ^∗∗^*p* = 0.001 versus mild group. (c) ESR levels are significantly different between all groups, apart from mild versus moderate (*p* = 0.98) and moderate versus severe groups (*p* = 0.06). ^∗^*p* < 0.05 versus mild, moderate, and severe groups. ^∗∗^*p* < 0.05 versus control and severe groups. ^#^*p* < 0.05 versus control. ^§^*p* < 0.05 versus control and mild groups. (d) UA levels are significantly elevated in severe compared to control, mild, and moderate groups. ^∗^*p* < 0.001 versus severe group. ^∗∗^*p* < 0.001 versus severe group. ^#^*p* < 0.05 versus severe group. ^§^*p* < 0.05 versus control, moderate, and severe groups.

**Figure 4:**
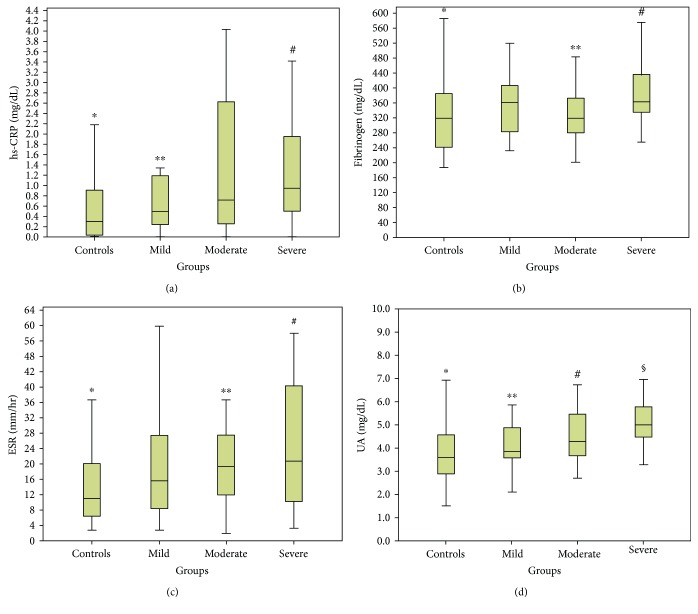
Serum high-sensitivity CRP (hs-CRP), fibrinogen, erythrocyte sedimentation rate (ESR), and uric acid (UA) in females according to OSA (obstructive sleep apnea) severity. (a) Serum hs-CRP is found elevated in severe compared to control and mild OSA patients. ^∗^*p* < 0.001 versus severe group, ^∗∗^*p* < 0.05 versus severe group, ^#^*p* < 0.05 versus control and mild groups. (b) serum fibrinogen is found elevated in severe compared to control and moderate OSA patients. ^∗^*p* = 0.01 versus severe group, ^∗∗^*p* = 0.026 versus severe group, ^#^*p* < 0.01 versus control and moderate groups. (c) ESR levels are significantly elevated in controls compared to moderate and severe groups. ^∗^*p* < 0.05 versus moderate and severe groups. ^∗∗^*p* = 0.02 versus controls. ^#^*p* < 0.001 versus controls. (d) UA levels are significantly different between all groups, apart from mild versus moderate group (*p* = 0.15). ^∗^*p* < 0.05 versus mild, moderate, and severe groups. ^∗∗^*p* < 0.05 versus control and severe groups. ^#^*p* < 0.05 versus control and severe groups. ^§^*p* < 0.01 versus control, mild, and moderate groups.

**Figure 5:**
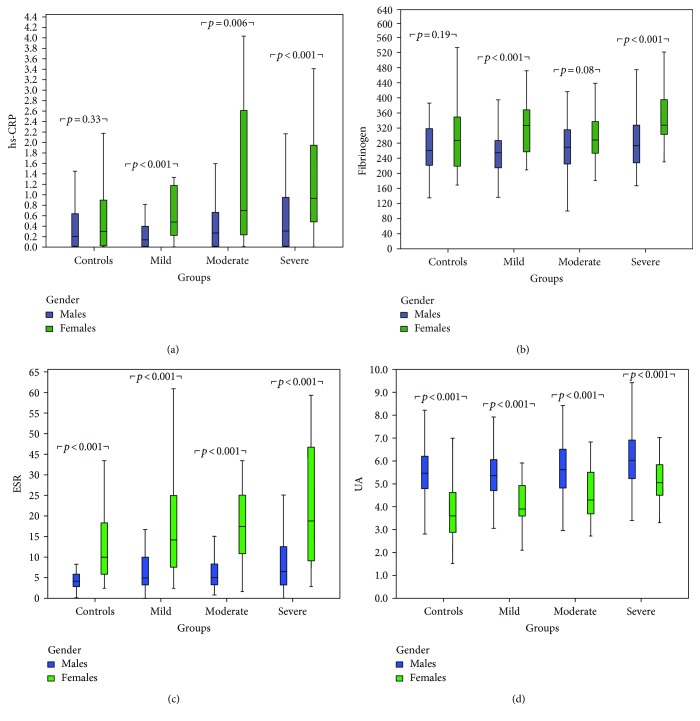
Serum high-sensitivity CRP (hs-CRP) (a), fibrinogen (b), erythrocyte sedimentation rate (ESR) (c), and uric acid (UA) (d) according to gender and OSA severity.

Since gender was among the strongest correlated factors in all four biomarkers, we divided the patients into male and female groups to eliminate this confounding factor. Females were older (43.7 ± 13.3 versus 41.6 ± 12.1, *p* = 0.017) with higher BMI (34.3 (15) versus 30.1 (6), *p* < 0.001) compared to males; meanwhile, no difference was noted in the percentage of active smokers (34.5% versus 40%, *p* = 0.11) between these two groups. In the entire cohort, females had increased levels of hs-CRP (0.69 versus 0.25, *p* < 0.001), fibrinogen (333.0 versus 299.1, *p* < 0.001), and ESR (18 versus 6, *p* < 0.001) compared to men. In contrast, UA levels were elevated in males compared to females (5.6 versus 4.2, *p* < 0.001). Then, patients' results were divided into 4 groups according to the OSA severity (Figures 3 and 4). Statistically significant changes in all biomarkers between groups were noted in both males and females (*p* < 0.05). Importantly, hs-CRP, fibrinogen, and ESR were significantly elevated in women compared to men in almost all OSA groups (*p* < 0.05), in contrast with UA which was significantly elevated in men in all OSA groups (*p* < 0.001) (Figure 5).

Finally, we evaluated the relationship between the four biomarkers and demographics and PSG parameters separately for men and women. Significant correlations were observed, with all biomarkers still showing a strong association with BMI (*p* < 0.001), AHI, ODI, and mean and min SaO2 (*p* < 0.01) in both gender groups. In males, stepwise multiple regression showed that after adjustment for confounders hs-CRP was still associated only with TST90 (*p* < 0.001), fibrinogen with AHI (*p* = 0.013) and ODI (*p* < 0.001), and ESR with ODI (*p* = 0.006) and mean SaO2 (*p* = 0.04). In females, hs-CRP (*p* < 0.001), fibrinogen (*p* < 0.001), and ESR (*p* < 0.001) were associated only with BMI. UA was still associated with ODI (*p* = 0.042) in men and min SaO2 (*p* = 0.042) in females.

### 3.5. Subgroup Analysis by BMI

Of the 1053 subjects, 116 (11%) had normal BMI, 363 (34.5%) were overweight, and 574 (54.5%) were obese. As BMI was also among the strongest correlated factors in all four biomarkers, we analyzed separately and according to OSA severity the subgroup of OSA patients with normal BMI in order to eliminate this confounding factor. Using AHI scores for classification of severity resulted in 66 controls (57%), 24 subjects with mild OSA (21%), 15 with moderate (13%), and only 11 with severe OSA (9%).

Age, gender, and BMI were significantly different between the four groups (*p* < 0.05), but no significant difference was found in smoking status (*p* > 0.05). There were no significant differences between all groups for hs-CRP, fibrinogen, and UA (*p* > 0.05). However, there was a statistically significant difference between groups in median ESR levels (*p* = 0.002). Significant correlations were observed, with hs-CRP showing association only with min SaO2 (*p* = 0.009) and TST90 (*p* = 0.001); fibrinogen with AHI (*p* = 0.003), ODI (*p* = 0.004), SaO2 mean (*p* = 0.008), and age (*p* = 0.022); and UA with SaO2 min (*p* = 0.004), male gender (*p* < 0.001), and BMI (*p* = 0.005). ESR was associated only with TST90, but close to the margin of statistical significance (*p* = 0.076). Stepwise multiple regression showed that after adjustment for confounders hs-CRP was still associated only with TST90 (*p* = 0.001), fibrinogen only with AHI (*p* = 0.003), and UA only with male gender (*p* < 0.001).

## 4. Discussion

OSA has been increasingly implicated in cardiovascular and cerebrovascular diseases [30–32]. The pathophysiologic mechanisms underlying these relationships remain unclear, but there is evidence to implicate vascular inflammation. Low-grade inflammation as a mechanism of cardiovascular alteration has been largely studied in OSA patients, with inflammatory biomarkers as particular targets. In order to investigate how early inflammatory mechanisms may be altered in OSA, we evaluated the association between OSA and levels of four circulating inflammatory biomarkers in a large population of consecutively enrolled subjects with a clinical suspicion of OSA. The current study recruited only those OSA patients who were newly diagnosed, treatment naive, and without any chronic or acute comorbidities; therefore, the elevated levels of plasma hs-CRP, fibrinogen, and UA in severe OSA patients reported were not caused by their presence. Interestingly, our results indicate that females diagnosed with OSA had higher levels of hs-CRP, fibrinogen, and ESR than men; conversely, men had higher UA levels than females in all groups of OSA severity.

We subsequently demonstrated important associations between biomarker levels and gender, BMI, and measures of OSA severity such as AHI, ODI, and mean and min SaO_2_. As inflammation is now recognized to be a characteristic of OSA [33], our finding of increased plasma levels in hs-CRP, fibrinogen, and UA in severe OSA patients was not unexpected. In addition, these biomarkers along with ESR were strongly correlated with AHI, independently of confounding factors, such as gender, BMI, and age, reinforcing findings from other studies that demonstrate a robust relationship between these biomarkers and OSA severity.

Hs-CRP has been the most studied inflammatory protein to date and a frequently used marker to predict the occurrence of cardiovascular diseases (CVDs). A number of studies have demonstrated that hs-CRP is a significant risk factor for atherosclerosis, and higher hs-CRP levels, even in the high normal range (0.2 to 1.5 mg/dL), are associated with high cardiovascular morbidity and mortality in individuals without known CVD [34–37]. Several authors have studied the relationship between AHI and hs-CRP levels in OSA patients, but the results are contradictory, possibly due to the role of obesity [6, 17, 33, 38–42]. Obesity indeed represents a strong confounding factor that makes difficult to study the respective implications of OSA severity in inflammatory biomarker alterations. However, in a recent meta-analysis, patients with OSA had a statistically significant higher level of CRP and this effect was positively influenced by OSA severity [43]. We found a relationship between OSA severity and hs-CRP concentrations in OSA patients without comorbidities. This relationship, although attenuated, remained significant after adjusting for BMI and gender. Furthermore, we analyzed separately the subgroup of OSA patients with normal BMI in order to eliminate the effect of BMI. Although, there were no significant differences between all groups for hs-CRP, fibrinogen, and UA, these results should be interpreted with caution because of the small sample size in the OSA severity subgroups in normal BMI subjects (probably due to losing power, *β*-error). Nevertheless, hs-CRP was still associated with TST90 and fibrinogen with AHI.

OSA-induced hypoxic conditions as well as sleep architecture disturbances due to repeated arousals may be also factors contributing to elevated levels of plasma fibrinogen in OSA patients. Fibrinogen, an acute-phase protein synthesized from the liver in response to infection and inflammation, is emerging as an important biomarker for cardiovascular risk [44, 45]. A recently published systemic review has confirmed the significance of elevated fibrinogen for prediction of future cardiovascular risk even in the healthy, middle-aged population [46]. Fibrinogen levels are increased in OSA patients [25, 47, 48], even after adjusting for comorbidities such as arterial hypertension or coronary artery disease, implicating hypercoagulability as a specific underlying OSA mechanism [20]. In a recent study, AHI was associated with CRP and fibrinogen, but these associations were substantially attenuated after adjustment for BMI and comorbidities [11]. In our study, OSA severity, as evidenced by oxygen desaturation index during sleep, was associated with increased plasma fibrinogen level independent of age, BMI, and active smoking, which is in accordance with a previous study [21]. By contrast, in one small study, hs-CRP and fibrinogen were not altered in OSA patients [42].

Other markers of inflammation, such as ESR are less well studied. The ESR, a cheap and widely used test, measures the tendency of red blood cells to aggregate. It is elevated in many of the acute and chronic inflammatory diseases [23] and has a possible predictive value for cardiovascular disease [49]. However, there are not enough data concerning the correlation between the severity of OSA and plasma ESR levels. Levels of ESR, hs-CRP, and fibrinogen were all found to be significantly higher in OSA patients compared to controls [25]. Min and colleagues suggested that there may be a positive association between ESR levels and nocturnal oxygen desaturation, although profound only in obese OSA patients [24]. In the current study, the linear regression model presented showed important correlation between AHI, ODI, and minimum SaO_2_ with ESR levels, even after controlling for confounders such as gender and BMI.

Finally, UA has been linked to OSA mainly through oxidative stress as a common pathway. Recurrent hypoxia, associated with OSA, leads to an increase in the degradation of adenosine triphosphatase into xanthine, which in turn increases uric acid concentrations [50]. Hyperuricemia is strongly associated with cardiovascular disease in OSA patients [51]. Previous studies observed an association between the presence of OSA and increased serum uric acid levels [26, 27, 52–54]. In a representative, large sample of the population of Sao Paulo, a strong association was found between UA levels and OSA, an association that remained significant even after adjustment for confounding factors such as gender, age, and BMI [12], in accordance with our results. Furthermore, AHI shows a significant linear relationship with UA [55]. It is worth noting that recently Kosacka and colleagues found that OSA patients with increased UA concentration have a higher risk of atherosclerosis, as indicated by a higher level of soluble proatherogenic ligand CD40, and a higher prevalence of cardiovascular adverse events [56].

Previous studies that evaluated the association of OSA with markers of inflammation were more prone to selection bias than the current study, and most of them included few or no women. The current study evaluates the association between treatment naive OSA patients, without comorbidities and markers of inflammation. Furthermore, we included a relatively large number of women. Gender differences in systemic inflammation in relation to OSA have been previously reported. Higher CRP levels have been found in women compared to men [57], probably due to a greater degree of adiposity in women. Importantly, we also found that in females, hs-CRP, fibrinogen, and ESR were significantly elevated in women compared to men in almost all OSA groups, in contrast with UA which was significantly elevated in males. In males, hs-CRP, fibrinogen, and ESR were associated with indices of OSA severity, after adjustment for confounders; however, in females, the same biomarkers were associated independently only with BMI. UA was still associated independently with OSA severity in both males and females. Previously, we have shown that female patients with moderate to severe OSA had higher although not statistically significant CRP values compared to matched males [58], and Min et al. showed increased ESR in female OSA patients [24]. More recently, Yardim-Akaydin et al. [59] showed statistically significant increased values of CRP, fibrinogen, and ESR in female OSA patients. In the present study, although there were more males in our patient group, the female gender seems to be more connected with increased values of inflammatory biomarkers.

There are some limitations of the current study that deserve comment. Firstly, the nature of cross-sectional design could not allow us to draw causal relationship between OSA and inflammatory biomarkers. Secondly, there were differences between the OSA patients and control subjects, as the OSA patients were older, mostly male and more obese; nevertheless, we performed adjustment for these significant clinical variables in our analysis. However, despite extensively adjusted for potential confounding variables, unrecognized biases regarding our findings still could not be ruled out. Thirdly, the study population consisted of patients without comorbidities; thus, the study results should be interpreted with caution in patients beyond this particular group.

Given the high prevalence of individuals with OSA and the serious health consequences of untreated OSA, it is likely that detection of OSA-induced subclinical disease, such as vascular inflammation revealed in our study, before the appearance of symptoms, may be of value in initial screening for OSA adding to pretest probability and therefore to referrals for evaluation. Furthermore, inflammatory biomarker levels may be involved in OSA severity and its cardiovascular comorbidities and should be considered in sleep apnea management in the future. As healthcare providers should be more aware of the significant health issues associated with OSA, a simple, timely blood test to identify individuals with suspected OSA and related cardiovascular consequences is of critical importance.

## 5. Conclusion

In conclusion, increased values of systemic inflammatory markers and their correlation with OSA severity observed in our study suggest the independent involvement of inflammation in OSA. However, the influence of gender should be taken into account. These findings may have important implications regarding OSA screening, diagnosis, treatment monitoring, and prognosis.

## Figures and Tables

**Table 1:** Characteristics of patients with obstructive sleep apnea (OSA) and controls.

Variables	Controls	Mild	Moderate	Severe	*p* value	*p* value	*p* value	*p* value	*p* value	*p* value	*p* value
					Across all	Controls versus mild	Controls versus moderate	Controls versus severe	Mild versus moderate	Mild versus severe	Moderate versus severe
Age (years)	38.8 ± 14.1	40.0 ± 11.8	43.4 ± 11.5	44.2 ± 11.1	<0.001	0.77	0.001	<0.001	0.013	<0.001	0.86
Gender (males, %)	53.7	77.1	75.5	84.9	<0.001	<0.001	<0.001	<0.001	0.69	0.013	0.004
BMI (kg/m2)	26.6 (6)	29.1 (6)	30.8 (7)	34.3 (11)	<0.001	<0.001	<0.001	<0.001	<0.001	<0.001	<0.001
Normal (18.5–24.9), *n* (%)	33.7	10.0	6.5	2.9	<0.001	<0.001	<0.001	<0.001	0.16	<0.001	0.03
Overweight (25–29.9), *n* (%)	42.1	55.5	35.3	19.6	<0.001	0.05	0.16	<0.001	<0.001	<0.001	<0.001
Obese (≥30), *n* (%)	24.2	38.1	58.2	77.3	<0.001	0.002	<0.001	<0.001	<0.001	<0.001	<0.001
Current smokers (%)	34.2	34.5	39.5	43.3	0.074	0.95	0.27	0.036	0.26	0.028	0.35
Former smokers (%)	18.4	20.2	21.5	29.2	0.008	0.65	0.44	0.005	0.73	0.012	0.033
Never smokers (%)	47.4	45.4	39.1	27.4	<0.001	0.68	0.09	<0.001	0.17	<0.001	0.003
Pack years	10 (28)	12 (25)	14 (30)	18 (35)	<0.001	0.75	0.42	<0.001	0.51	<0.001	0.002
ESS	7 (9)	8 (8)	9 (9)	12 (9)	<0.001	0.93	0.003	<0.001	0.002	<0.001	0.002
AHI (/h)	2 (2)	10 (6)	21 (6)	57 (34)	<0.001	<0.001	<0.001	<0.001	<0.001	<0.001	<0.001
ODI (/h)	2 (2)	9 (5)	20 (8)	59 (34)	<0.001	<0.001	<0.001	<0.001	<0.001	<0.001	<0.001
AI (/h)	24 (14)	31 (14)	34 (18)	46 (25)	<0.001	<0.001	<0.001	<0.001	0.04	<0.001	<0.001
Mean SaO_2_	96 (2)	95 (1)	94 (2)	92 (39)	<0.001	<0.001	<0.001	<0.001	<0.001	<0.001	<0.001
Min SaO_2_	92 (3)	88.5 (4)	85 (5)	79 (66)	<0.001	<0.001	<0.001	<0.001	<0.001	<0.001	<0.001
TST90 (min)	0 (0)	1 (3)	9 (11)	69 (101)	<0.001	<0.001	<0.001	<0.001	<0.001	<0.001	<0.001

BMI: body mass index; ESS: Epworth sleepiness scale; AHI: apnea-hypopnea index; AI: arousal index; ODI: oxygen desaturation index; SaO_2_: oxygen saturation; TST90: sleep time spent with SaO2 less than 90%.

**Table 2:** Percentage of subjects with high inflammatory biomarkers in the four OSA severity groups.

Variables	Controls	Mild	Moderate	Severe	*p* value	*p* value	*p* value	*p* value	*p* value	*p* value	*p* value
					Across all	Controls versus mild	Controls versus moderate	Controls versus severe	Mild versus moderate	Mild versus severe	Moderate versus severe
Hs-CRP (≥1 mg/dL)	23%	17.2%	27.7%	28.2%	0.04	0.2	0.36	0.26	0.02	0.008	0.9
Fibrinogen (>400 mg/dL)	11.8%	13.4%	10.1%	19.8%	0.04	0.7	0.68	0.07	0.39	0.11	0.016
ESR (>30 mm/hr)	7.8%	7.5%	11.1%	11.7%	0.36	0.91	0.33	0.21	0.25	0.14	0.84
UA (>7 mg/dL)	6.8%	8.6%	13.7%	17.5%	0.003	0.51	0.036	0.001	0.14	0.008	0.32

hs-CRP: hs-C-reactive protein; ESR: erythrocyte sedimentation rate; UA: uric acid.
